# Nonlinear Complementary Filter for Attitude Estimation by Fusing Inertial Sensors and a Camera

**DOI:** 10.3390/s20236752

**Published:** 2020-11-26

**Authors:** Lingxiao Zheng, Xingqun Zhan, Xin Zhang

**Affiliations:** School of Aeronautics and Astronautics, Shanghai Jiao Tong University, Shanghai 200240, China; lx_zheng@sjtu.edu.cn (L.Z.); xin.zhang@sjtu.edu.cn (X.Z.)

**Keywords:** consumer electronics, attitude estimation, inertial sensors, camera, nonlinear complementary filter

## Abstract

Using a standalone camera for pose estimation has been quite a standard task. However, the point correspondence-based algorithms require at least four feature points in the field of view. This paper considers the situation that there are only two feature points. Focusing on the attitude estimation, we propose to fuse a camera with low-cost inertial sensors based on a nonlinear complementary filter design. An implicit geometry measurement model is derived using two feature points in an image. This geometry measurement is fused with the angle rate measurement and vector measurement from inertial sensors using the proposed nonlinear complementary filter with only two parameters to be adjusted. The proposed nonlinear complementary filter is posed directly on the special orthogonal group SO(3). Based on the theory of nonlinear system stability analysis, the proposed filter ensures locally asymptotic stability. A quaternion-based discrete implementation of the filter is also given in this paper for computational efficiency. The proposed algorithm is validated using a smartphone with built-in inertial sensors and a rear camera. The experimental results indicate that the proposed algorithm outperforms all the compared counterparts in estimated accuracy and provides competitive computational complexity.

## 1. Introduction

Attitude estimation using low-cost sensors plays an important role in many consumer electronic applications and has attracted much research attention. For example, in rehabilitation and biomedical engineering, the attitude information is applied for elderly fall detection [1]. In indoor pedestrian dead reckoning application, the attitude of the measurement unit is used for step detection and heading estimation [2].

MARG (magnetic, angular rate, and gravity) sensors and monocular cameras are two kinds of low-cost sensors that are widely used in consumer electronic applications to provide attitude information [3,4,5]. However, using MARG sensors or camera standalone for attitude estimation has some limitations.

On the one hand, the MARG sensors contain a magnetometer which is used to correct heading drift of the attitude estimation. Generally speaking, the magnetometer is factory calibrated to compensate for any error sources that are internal to the device. However, for the errors that are introduced externally by mounting structures or adjacent devices, an additional calibration process is essential [6]. In order to calibrate the magnetometer, the device needs to be moved in all possible directions to collect data. This is not user-friendly. Moreover, when it works in an environment with abnormal magnetic fields, the attitude estimation performance will deteriorate significantly.

On the other hand, when there are artificial vision fiducials arranged in the environment, the attitude and position of a camera can be recovered from one image by solving the perspective-and-point (PnP) problem [7,8]. The point correspondence-based algorithms require at least four feature points in the field of view. However, in a dynamic and possibly cluttered environment, the number of feature points may be less than four.

The goal of this work is to propose a low-cost fusion method to achieve absolute attitude estimation in an environment with only two pre-calibrated artificial vision fiducials. The sensors to be fused include gyroscope, accelerometer, and camera. The advantages of such a sensor combination are twofold:

- Compared with the MARG sensor combination, our method can work in an abnormal magnetic field environment, especially in an indoor environment.
- Compared with the combination of gyroscope and camera, our method still converges when there are only two feature points in the image.

The second one is important for the resource-constrained artificial fiducial system, such as the AprilTag system [9] and the visible light communication reference system [10,11].

The main contributions of our work are summarised as:

- We derive an implicit geometry measurement for camera-based attitude estimation. This measurement is associated with attitude and is independent of the position of the camera.
- A nonlinear complementary filter is proposed to fuse angle rate measurement, vector measurement, and geometry measurement. There are only two parameters to be adjusted.

The remainder of this paper is organized as follows. Section 2 explores the literature of attitude estimation algorithms based on MARG sensors, camera standalone, and visual-inertial fusion, respectively. Section 3 presents the sensor models including angle rate measurement, vector measurement, and the proposed geometry measurement model. Section 4 presents the nonlinear complementary filter fusing inertial sensors and a camera. The stability analysis of the proposed filter is in this section. An attitude initial alignment method is proposed to provide the initial value of the filter. The discrete implementation of the filter on quaternion is also given in this section. The algorithms are validated using data collected by a smartphone with built-in inertial sensors and a rear camera. Three other representative methods of attitude estimation algorithms are also implemented on the collected data. The results are shown in Section 5. Finally, concluding remarks and future work are presented in Section 6.

## 2. Related Works

Various solutions have been proposed for attitude estimation using low-cost sensors, including (1) MARG (magnetic, angular rate, and gravity) sensors-based methods, (2) monocular camera-based methods.

For MARG sensors-based methods, the gyroscope provides angle rate measurement. The accelerometer and magnetometer provide attitude associated vector measurements. When the initial attitude is known, attitude can be computed by integrating the angle rate measurement [12]. Meanwhile, the attitude can be constructed directly from the vector measurements [13]. To estimate the attitude from vector measurements is to solve a least square problem, the Wahba’s problem. A unique closed-form solution can be provided by the QUEST (quaternion estimator) algorithm in [14] and singular value decomposition (SVD)-based method in [15].

The attitude estimation accuracy obtained by numerical integration of the angle rate measurement is good in a short time. However, the bias and noise of the gyroscope make the estimated value deviate more and more from the true value over time. On the other hand, the attitude recovered from vector measurements hosts long-term stability. However, the instantaneous linear acceleration and magnetic field anomalies will decrease the estimation accuracy.

To achieve good bandwidth and long-term stability, many MARG sensors-based attitude estimation algorithms resort to fuse the angle rate measurement and the vector measurements. The classic fusion algorithms are based on the extended Kalman filter [16,17]. These stochastic approaches involve the update of the error covariance matrix and gain matrix which will lead to a large computational burden.

The Mahony’s nonlinear complementary filter formulates the fusion problem as deterministic nonlinear observer kinematics on the special orthogonal group [18]. The observer kinematics include a prediction term based on the angle rate measurement and a correction term derived from the estimation residual. To calculate the correction term, the direct and passive versions of Mahony’s complementary filter rely on the algebraic reconstruction of attitude from vector observations, while the explicit version explicitly uses the cross product of the reference vectors and the observed vectors. Mahony’s complementary filter has only two adjustable parameters and ensures almost global asymptotic stability. Two passive nonlinear complementary filters algorithms that are implemented on quaternion are proposed in [19,20]. The algebraic reconstruction of attitude from vector observation is based on Levenberg–Marquardt optimization algorithm in [19] and singular value decomposition (SVD) in [20], respectively.

Different from the nonlinear complementary filter, the linear complementary filter linearly combines the attitude quaternion integrated from the angular velocity with the one reconstructed from the vector observations. Therefore, the linear complementary filter has a frequency domain interpretation. The Madgwick’s linear complementary filter in [21] applies the gradient descent algorithm to solve the quaternion version of Wahba’s problem. The optimization algorithm only computes one iteration per time sample, provided that the convergence rate of the estimated attitude is equal to or greater than the change rate of physical orientation. In [22], a fast complementary filter is proposed by deriving a quaternion increment that is free of iterations. An improved gradient descent based attitude complementary filter in [23] provides fast error convergence and robustness by decoupling the magnetic field variance from roll and pitch. Gain-scheduled or adaptive complementary filters are more robust to strong accelerations and magnetic field disturbances than gain-fixed complementary filters [4,24].

For attitude estimation, in addition to MARG sensors, the monocular camera is also an attractive low-cost sensor. Using a camera standalone for pose estimation is quite a standard task. When there are artificial vision fiducials arranged in the environment, the attitude and position of the camera can be recovered from one image by solving the PnP problem [7,8]. In an environment without artificial fiducials, the relative rotation and scaled translation can be restored from two images with natural features. This problem has been extensively researched, and a large number of algorithms have been developed. The most well-known ones are the 8-point algorithm [25] and the 5-point minimal algorithm [26].

Considering that the frame rate of the low-cost camera is relatively low, it is expected to fuse inertial sensors and a camera to get a higher data rate. The monocular visual-inertial system (VINS) based on extended Kalman filter [27] or bundle adjustment formulation [28] can provide relative attitude estimation. However, VINS or standalone camera pose estimation simultaneously calculates the attitude and position of the camera. When the attitude is the only one to be interested, position and attitude should be decoupled to avoid unnecessary calculations.

Recently, a generalized linear complementary filter for attitude estimation from multi-sensor measurements is proposed in [29]. The point-correspondence constraints of the camera are modeled as vector measurements. This model allows the camera to be fused with a gyroscope in the same way as an accelerometer and a magnetometer. However, to satisfy the premise of vector measurement, the position of the camera must be close enough to the origin of the reference coordinate system.

An implicit measurement model proposed in [30] enables a strict decoupling of attitude and position. The measurements of the camera are fused with angle rate measurement using a nonlinear observer. However, this implicit measurement model is based on line-correspondences instead of point-correspondence. Compared with the point feature, tracking the line feature is computationally more intensive.

In this paper, we use two feature points to derive an implicit geometry measurement that has the same expression as the implicit measurement in [30], but without tracking the line-correspondences. The new geometry measurement is fused with the vector measurement from accelerometers and the angle-rate measurement from gyroscopes. This combination of sensors makes it possible to determine the absolute attitude with only two feature points in the field of view. Meanwhile, the fusion of these three kinds of measurements removes the restrictions on the position of the camera in [29].

## 3. Sensor Models

This section presents the sensor measurement models for attitude estimation. The angle-rate measurement and vector measurement from gyroscopes and accelerometers are briefly described. The camera geometry measurement that is associated with the attitude but is independent of the position of the camera is derived in detail.

### 3.1. Inertial Sensors

In this paper, the body-fixed frame of reference {b} is a right-forward-up coordinate system. The navigation frame of reference {n} is an east-north-up coordinate system. The direction cosine matrix $C_{b}^{n}$ denotes the relative orientation of {b} with respect to {n}. To avoid the repeated occurrence of superscript and subscript, we use R to represent $C_{b}^{n}$. R and $C_{b}^{n}$ belong to special orthogonal group denoted by SO(3).

The measurements available from inertial sensors are 3 axis gyroscopes and 3 axis accelerometers.

Gyroscopes measure the angle rate of {b} relative to {n} expressed in {b}. We assume that the initial bias of the gyroscope has been calibrated in the initial static stage and is subtracted from the gyroscope measurement. Therefore, the angle-rate measurement model for a low-cost gyroscope is

(1) $$\tilde{\omega }=\omega +\mu_{\omega }$$

where ω denotes the true angle rate, $\mu_{\omega }$ denotes the additive error. It should be noticed that the earth rotation angle rate is submerged in error $\mu_{\omega }$ for low-cost gyroscopes [31].

The kinematics of the true system that describe the relationship between attitude R and angle rate ω is

(2) $$\dot{R}=R\omega \times$$

where (·)× donates the skew-symmetric matrix form of the preceding vector

(3) $$\omega \times =\left[\begin{array}{ccc} 0 & -\omega_{3} & \omega_{2}\\ \omega_{3} & 0 & -\omega_{1}\\ -\omega_{2} & \omega_{1} & 0 \end{array}\right]$$

Accelerometers provide the measurement of “up” acceleration against the earth’s gravity. The measurement model of the corresponding “vector measurement” [13,29] is

(4) $$\tilde{a}=R^{T}e_{3}+\mu_{a}$$

where $e_{3}={\left[\begin{array}{ccc} 0 & 0 & 1 \end{array}\right]}^{T}$ is the reference vector of “up” in the navigation frame. $\tilde{a}$ is the normalized measurement vector in the body frame {b} from the accelerometer. $\mu_{a}$ is the error term caused by the measurement noise and the potential linear acceleration in which the latter one is small and fast, varying about zero. That is to say, the gravity dominates the value of $\tilde{a}$ for sufficiently low frequency response. $R^{T}=C_{n}^{b}$ is the coordinate transformation matrix that can be used to transform the components of a vector from {n} into {b}.

### 3.2. Monocular Camera

**Figure 1:**
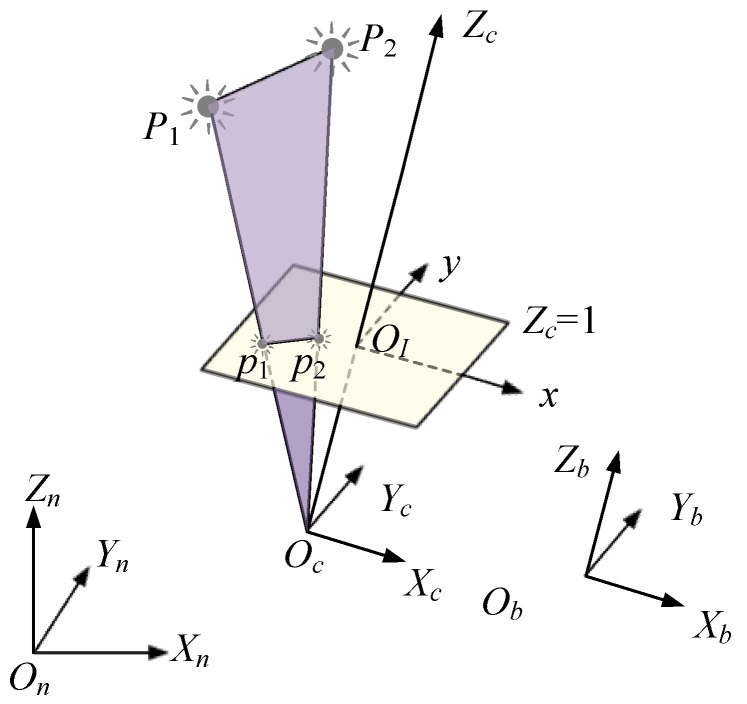
The frames of reference and camera model.

We assume that the reference frame of the camera {c} is aligned with the body frame so that $C_{c}^{n}=C_{b}^{n}=R$. The z-axis of {c} is the optical axis shown in Figure 1. The geometric relationship between the origins of {c} and {n} and any point P in the 3D world satisfies vector addition $\vec{O_{c}P}=\vec{O_{c}O_{n}}+\vec{O_{n}P}$. Using this geometric relationship, an observation of the attitude and position of the camera can be achieved as

(5) $$P^{\left\{c\right\}}=t+R^{T}P^{\left\{n\right\}}$$

where $P^{\left\{c\right\}}$ and $P^{\left\{n\right\}}$ are the 3D coordinates of point P expressed in the camera frame and navigation frame. t is a translation vector. The components of t are equal to the coordinates of $O_{n}$ in camera frame {c}.

As seen from (5), if t is a zero vector, the normalized $P^{\left\{c\right\}}$ and $P^{\left\{n\right\}}$ can be used as a vector measurement for attitude estimation. However, this assumption is not always feasible in practice. To avoid restrictions on camera translation, we use two point-correspondences to derive a geometry measurement. No translation vector t is in the new measurement model.

As shown in Figure 1, *P*_1_ and *P*_2_ are two artificial feature points that can be captured by a camera. Considering that the camera is modeled by a perspective camera using the “frontal projection model” [32], *p*${}_{1}$ and *p*${}_{2}$ are the projections on normalized image plane $Z_{c}=1$.

Let $P_{1}^{\left\{c\right\}}$ and $P_{2}^{\left\{c\right\}}$ denote the coordinates of feature points in frame {c}. Let $p_{1}$ and $p_{2}$ denote the normalized image coordinate values obtained from the image. A homogeneous model describing the relationship between $p_{i}$ and $P_{i}^{\left\{c\right\}}$ is

(6) $$p_{i}={\left[\begin{array}{ccc} \frac{P_{i,x}^{\left\{c\right\}}}{P_{i,z}^{\left\{c\right\}}} & \frac{P_{i,y}^{\left\{c\right\}}}{P_{i,z}^{\left\{c\right\}}} & 1 \end{array}\right]}^{T}+\mu_{pi},\;i=1,2$$

where $\mu_{pi}$ is the error of the projection model. $P_{i,z}^{\left\{c\right\}}$ is the so-called “depth” of a feature point in the camera frame.

The plane formed by the camera center point and two feature points is defined as the “feature plane” in this paper, as shown with light purple color in Figure 1. Let $\tilde{y}$ denote the computed unit normal vector of the feature plane expressed in {c}. $\tilde{y}$ is calculated by

(7) $$\tilde{y}=\frac{p_{1}\times p_{2}}{norm(p_{1}\times p_{2})}$$

Let d denote the true unit normal vector of the feature plane expressed in {n}. Let r denote the unit direction vector of $\vec{P_{1}P_{2}}$ expressed in {n}.

The explicit error model of the proposed geometry measurement is

(8) $$\tilde{y}=R^{T}d+\mu_{y}$$

and the implicit model of geometry measurement is

(9) $${\tilde{y}}_{}^{T}R^{T}r=0+\mu_{y}^{\prime }$$

where $\mu_{y}$ and $\mu_{y}^{\prime }$ are the measurement errors that come from the errors in $p_{1}$ and $p_{2}$.

The positions of artificial feature points in frame {n} can be pre-calibrated in the offline stage. Therefore, r is a known reference vector, and the implicit geometry measurement in (9) can be used for attitude estimation. The explicit geometry measurement model in (8) is not useless in this paper. It appears in the stability analysis of the attitude observer in Section 4.2.

## 4. Attitude Estimation Algorithm

In this section, a nonlinear complementary filter on the special orthogonal group is introduced. This filter fuses the angle-rate measurement, vector measurement, and implicit geometry measurement to estimate the attitude in continuous dynamics. The attitude estimation algorithm for real-world signals is also considered in this section. Specifically, it includes the initial alignment and the discrete realization of the filter based on the unit quaternion.

### 4.1. Nonlinear Complementary Filter on SO(3)

The proposed attitude estimation algorithm is based on a nonlinear observer. The goal of the attitude estimation observer is to provide a set of dynamics for an estimated attitude to drive the estimation error converges.

The observer kinematics include a prediction term and a correction term. In this paper, the prediction term is based on the angle rate measurement. The correction term is added to the measured angle rate as it does in classic attitude observer design [18,30].

Let $\hat{R}$ denote the estimated direction cosine matrix $C_{b}^{n^{\prime }}$. The proposed attitude observer is

(10) $$\dot{\hat{R}}=\hat{R}\left(\tilde{\omega }+\Delta \omega \right)\times ,\;\hat{R}\left(0\right)={\hat{R}}_{0}$$

where the correction term Δω is

(11) $$\begin{array}{c} \Delta \omega =k_{a}\Delta \omega_{a}+k_{c}\Delta \omega_{c}\\ \Delta \omega_{a}=\tilde{a}\times {\hat{R}}^{T}e_{3}\\ \Delta \omega_{c}=-{\tilde{y}}^{T}{\hat{R}}^{T}r\cdot \tilde{y}\times {\hat{R}}^{T}r \end{array}$$

$\Delta \omega_{a}$ is the correction term caused by the vector measurement. Referring to the explicit version of Mahony’s complementary filter [18], the cross product of the estimated vectors ${\hat{R}}^{T}e_{3}$ and the observed vectors $\tilde{a}$ is used to construct the correction term.

$\Delta \omega_{c}$ is the correction term caused by the geometry measurement. This correction term is referred from the observer design in [30] that fuses angle-rate measurement and geometry measurement. The rationale for the $\Delta \omega_{c}$ is the following. From the implicit geometry measurement Equation (9), the ideal $\hat{R}$ satisfies ${\hat{R}}^{T}r\in \ker\tilde{y}$. If it is not satisfied, then a corrective angle rate should be applied. The angle rate needed for this is directed along $-\tilde{y}\times {\hat{R}}^{T}r$. The magnitude of the correction is simply the ${\tilde{y}}^{T}{\hat{R}}^{T}r$.

In the above attitude observer, $k_{a}>0$ and $k_{c}>0$ are two fixed parameters. The stability of the new attitude observer is analyzed in Section 4.2.

As [18] does, we also term the observer as a nonlinear complementary filter here.

### 4.2. Stability Analysis

The estimation error $\tilde{R}$ is defined as the relative rotation from the true navigation frame {n} to the estimated navigation frame $\left\{n^{\prime }\right\}$, that is

(12) $$\tilde{R}:=R{\hat{R}}^{T},\;\tilde{R}=C_{n^{\prime }}^{n}$$

Differentiate both sides of estimation error definition Equation (12) and it is straightforward to verify that the error system is

(13) $$\begin{array}{cc} \dot{\tilde{R}} & =\dot{R}{\hat{R}}^{T}+R{\dot{\hat{R}}}^{T}\\ & =R\omega \times {\hat{R}}^{T}+R\left(\tilde{\omega }+\Delta \omega \right)\times^{T}{\hat{R}}^{T}\\ & =R\omega \times {\hat{R}}^{T}-R\left(\tilde{\omega }+\Delta \omega \right)\times {\hat{R}}^{T} \end{array}$$

Substitute the angle rate measurement $\tilde{\omega }$ using its true values ω. Using RΔω×=(RΔω)×R, we obtain:

(14) $$\begin{array}{cc} \dot{\tilde{R}} & =-R\Delta \omega \times {\hat{R}}^{T}\\ & =-\left(R\Delta \omega \right)\times R{\hat{R}}^{T}\\ & =-\left(R\Delta \omega \right)\times \tilde{R} \end{array}$$

Based on the correction term in (11), the error system is given by

(15) $$\begin{array}{cc} \dot{\tilde{R}} & =-k_{a}\left(R\Delta \omega_{a}\right)\times \tilde{R}-k_{c}\left(R\Delta \omega_{c}\right)\times \tilde{R}\\ & =-k_{a}\left(R\tilde{a}\times {\hat{R}}^{T}e_{3}\right)\times \tilde{R}\\ & \;+k_{c}{\tilde{y}}^{T}{\hat{R}}^{T}r\cdot \left(R\tilde{y}\times {\hat{R}}^{T}r\right)\times \tilde{R} \end{array}$$

Substitute the vector measurement $\tilde{a}$ and geometry measurement $\tilde{y}$ using their true values $R^{T}e_{3}$ and $R^{T}d$. Here, the explicit geometry measurement model is used. It is straightforward that

(16) $$\begin{array}{cc} \dot{\tilde{R}} & =-k_{a}\left[R\left(R^{T}e_{3}\right)\times {\hat{R}}^{T}e_{3}\right]\times \tilde{R}\;\\ & \;+k_{c}d^{T}R{\hat{R}}^{T}r\cdot \left[R\left(R^{T}d\right)\times {\hat{R}}^{T}r\right]\times \tilde{R} \end{array}$$

For any vector v, there is $\left(R^{T}v\right)\times =R^{T}v\times R$. Using this relationship, R is eliminated from the error dynamics. The error system is

(17) $$\begin{array}{cc} \dot{\tilde{R}} & =-k_{a}\left(e_{3}\times \tilde{R}e_{3}\right)\times \tilde{R}\\ & \;+k_{c}d^{T}\tilde{R}r\cdot \left(d\times \tilde{R}r\right)\times \tilde{R} \end{array}$$

The error system in (17) is a nonlinear time-varying system because the feature plane’s unit normal vector d is varying with the position of the camera.

It is easily verified that the identity matrix I is an equilibrium point of the error system. According to the definition of the small perturbing rotation [32], the attitude error around the equilibrium point ${\tilde{R}}_{eq}$ is approximated by

(18) $$\tilde{R}\approx \left(I+\phi \times \right){\tilde{R}}_{eq}$$

where vector ϕ is the angle-axis form of the small perturbing rotation.

The linearization of the error dynamics is computed to analyze the local stability of the equilibrium point [33]. Substitute (18) into (17) with ${\tilde{R}}_{eq}=I$, we get

(19) $$\begin{array}{cc} \dot{\phi }\times & \approx -k_{a}\left(e_{3}\times \phi \times e_{3}\right)\times \\ & +k_{c}d_{}^{T}\phi \times r\left(d\times r\right)\times \end{array}$$

Using the properties of linear operations, including the skew-symmetric matrix transformation and the vector cross product, the state equation describing the evolving of the ϕ is obtained as follows:

(20) $$\begin{array}{cc} \dot{\phi } & =k_{a}\left(e_{3}\times \right)\left(e_{3}\times \right)\phi \\ & -k_{c}\left(d\times r\right){\left(d\times r\right)}^{T}\phi \end{array}$$

For $k_{a}>0$, $k_{c}>0$, the linearized system is asymptotically stable as long as the third component of d×r is not 0. Otherwise, the error of the yaw angle will not converge to zero. According to the geometric relationship, d×r is the vector located in the feature plane and perpendicular to the line of two feature points. This geometric structure is determined by the positions of features and camera. Since the linearized error system is asymptotically stable, the nonlinear error system ensures locally asymptotic stability around the equilibrium point I.

The nonlinear complementary filter proposed in this paper is based on observer design. The local asymptotic stability of the filter ensures that the initial attitude estimation error will converge to zero when the gains are any constant greater than zero. Generally speaking, the larger the gain, the faster the error convergence. However, the observer design method is a deterministic method in which the measurement errors are assumed to be zero. The measurement noise in practice will lead to a big attitude estimation variance when the gains are tuned too large inappropriately. Therefore, gains tuning is a compromise process. Moreover, a proper initial attitude guess is important for the filter with local asymptotic stability.

### 4.3. Initial Alignment

The initial alignment is the problem of attitude determination in the initial static stage using accelerometers and a camera. It is important since the attitude estimation filter in (10) requires a proper initial value.

According to the chain rule, the direction cosine matrix’s transpose $C_{n}^{b}$ can be written as the multiplication of two rotation matrixes

(21) $$C_{n}^{b}=C_{h}^{b}C_{n}^{h}$$

where {h} is an intermediate frame system, referred to as the horizontal frame, whose z-axis coincides with the z-axis of {n}. Under the definition of the navigation frame and the body frame in this paper, $C_{h}^{b}$ and $C_{n}^{h}$ can be written in the following form:

(22) $$C_{h}^{b}=\left[\begin{array}{ccc} \cos\varphi & \sin\theta \sin\varphi & -\cos\theta \sin\varphi \\ 0 & \cos\theta & \sin\theta \\ \sin\varphi & -\sin\theta \cos\varphi & \cos\theta \cos\varphi \end{array}\right]$$

(23) $$C_{n}^{h}=\left[\begin{array}{ccc} \cos\psi & \sin\psi & 0\\ -\sin\psi & \cos\psi & 0\\ 0 & 0 & 1 \end{array}\right]$$

The $\left(\begin{array}{ccc} \theta & \varphi & \psi \end{array}\right)$ is a set of Euler angles. θ is the pitch angle with θ∈[−90°,90°). φ and ψ are the roll angle and yaw angle, where φ,ψ∈[−180°,180°). The yaw angle ψ equals to the angle formed by the y-axis of {h} and the y-axis of {n}.

The initial alignment method proposed in this paper includes horizontal alignment and azimuth alignment and solution confirmation.

First, use normalized accelerometer vector measurement to calculate the horizontal attitude. It is easily verified that the third column of $C_{h}^{b}$ is the projection of the normalized “up” vector in the body frame {b}. Therefore, $C_{h}^{b}$ can be recovered roughly from the acceleration measurement by following expressions

$$\begin{array}{cc} \sin\theta ={\tilde{a}}_{y}, & \cos\theta =\sqrt{1-{{\tilde{a}}^{2}}_{y}} \end{array}$$

$$\begin{array}{c} \sin\varphi =\left\{\begin{array}{cc} \frac{-{\tilde{a}}_{x}}{\sqrt{1-{\tilde{a}}_{y}^{2}}}, & {\tilde{a}}_{y}^{2}\neq 1\\ 0, & {\tilde{a}}_{y}^{2}=1 \end{array}\right. \end{array}$$

$$\begin{array}{c} \cos\varphi =\left\{\begin{array}{cc} \frac{{\tilde{a}}_{z}}{\sqrt{1-{\tilde{a}}_{y}^{2}}}, & {\tilde{a}}_{y}^{2}\neq 1\\ 1, & {\tilde{a}}_{y}^{2}=1 \end{array}\right. \end{array}$$

Second, determine the azimuth. After calculating of the $C_{h}^{b}$, use camera implicit geometry constraint

(24) $${\tilde{y}}^{T}C_{h}^{b}C_{n}^{h}r=0$$

to construct one linear equation about sinψ and cosψ. With the constraint $\sin^{2}\psi +\cos^{2}\psi =1$, two sets of solutions can be found, one of which can be confirmed by the constraints of the camera’s field of view.

Third, confirm the true direction cosine matrix. According to the camera model in (5) and (6), it is straightforward that

(25) $$C_{n}^{b}\left(P_{1}^{\left\{n\right\}}-P_{2}^{\left\{n\right\}}\right)=z_{1}p_{1}-z_{2}p_{2}$$

In (25), $z_{1}$ and $z_{2}$ are the depth of feature points P1 and P2 in the camera frame. Using (25), three linear equations about $z_{1}$ and $z_{2}$ can be obtained. The $C_{n}^{b}$ that leads to positive $z_{1}$ and $z_{2}$ will be accepted as the initial direction cosine matrix.

### 4.4. Discrete Implementation on Quaternion

The filter in (10) is a continuous system. In practical implementation, sensor data will be sampled and the filter needs to be integrated in discrete time. The unit quaternion representation of the rotations is commonly used for the realization of algorithms on SO(3) since it offers considerable efficiency in code implementation [18]. The proposed attitude observer in quaternion representation is

(26) $$\dot{\hat{q}}=\frac{1}{2}\Omega \left(\tilde{\omega }+\Delta \omega \right)\hat{q},\;\hat{q}\left(0\right)={\hat{q}}_{0}$$

where

(27) $$\Omega \left(\omega \right)=\left[\begin{array}{cccc} 0 & -\omega_{1} & -\omega_{2} & -\omega_{3}\\ \omega_{1} & 0 & \omega_{3} & -\omega_{2}\\ \omega_{2} & -\omega_{3} & 0 & \omega_{1}\\ \omega_{3} & \omega_{2} & -\omega_{1} & 0 \end{array}\right]$$

Since the term $\tilde{\omega }+\Delta \omega$ can be seen as the corrected angle rate in the frame of {b}, according to the quaternion-based attitude update algorithm [12], the discrete implementation of filter (26) is

(28) $${\hat{q}}_{k}={\hat{q}}_{k-1}{}^{\circ}\Delta {q_{\tilde{\omega }+\Delta \omega }}_{k}$$

∘ in (28) is the quaternion multiplication operator. p°q is defined by

(29) $$p{}^{\circ}q=\left[\begin{array}{c} p_{1}q_{1}-p_{2}q_{2}-p_{3}q_{3}-p_{4}q_{4}\\ p_{1}q_{2}+p_{2}q_{1}+p_{3}q_{4}-p_{4}q_{3}\\ p_{1}q_{3}+p_{3}q_{1}+p_{4}q_{2}-p_{2}q_{4}\\ p_{1}q_{4}+p_{4}q_{1}+p_{2}q_{3}-p_{3}q_{2} \end{array}\right]$$

$\Delta {q_{\tilde{\omega }+\Delta \omega ,}}_{k}$ in (28) is the quaternion increment from time $t_{k-1}$ to time $t_{k}$ and is calculated by

(30) $$\Delta {q_{\tilde{\omega }+\Delta \omega ,}}_{k}=\left[\begin{array}{c} \cos\frac{\Delta \theta_{k}}{2}\\ \frac{\Delta \theta_{k}}{\Delta \theta_{k}}\sin\frac{\Delta \theta_{k}}{2} \end{array}\right]$$

$\Delta \theta_{k}$ in (30) is the angle increment vector, $\Delta \theta_{k}=\left|\Delta \theta_{k}\right|$ and

(31) $$\Delta \theta_{k}=\left({\tilde{\omega }}_{k}+\Delta \omega_{k}\right)\Delta t$$

where Δt is the time interval between $t_{k-1}$ and $t_{k}$. ${\tilde{\omega }}_{k}$ is calculated by the average value of the gyroscope measurements in $t_{k-1}$ and $t_{k}$.

$\Delta \omega_{k}$ in angle increment vector (31) is the angle rate correction term constructed by the estimation in time $t_{k-1}$ and measurements in time $t_{k}$ as

(32) $$\begin{array}{c} \Delta \omega_{k}=k_{a}\Delta \omega_{a,k}+k_{c}\Delta \omega_{c,k}\\ \Delta \omega_{a,k}={{\tilde{a}}^{}}_{k}\times C\left({\hat{q}}_{k-1}\right)e_{3}\\ \Delta \omega_{c,k}=-{\tilde{y}}_{k}^{T}C\left({\hat{q}}_{k-1}\right)r\cdot {{\tilde{y}}^{}}_{k}\times C\left({\hat{q}}_{k-1}\right)r \end{array}$$

C(q) is the coordinate transformation matrix and equals to

$$\left[\begin{array}{ccc} q_{1}^{2}+q_{2}^{2}-q_{3}^{2}-q_{4}^{2} & 2(q_{2}q_{3}+q_{1}q_{4}) & 2(q_{2}q_{4}-q_{1}q_{3})\\ 2(q_{2}q_{3}-q_{1}q_{4}) & q_{1}^{2}-q_{2}^{2}+q_{3}^{2}-q_{4}^{2} & 2(q_{3}q_{4}+q_{1}q_{2})\\ 2(q_{2}q_{4}+q_{1}q_{3}) & 2(q_{3}q_{4}-q_{1}q_{2}) & q_{1}^{2}-q_{2}^{2}-q_{3}^{2}+q_{4}^{2} \end{array}\right]$$

To get a high-bandwidth system, the sample interval of the gyroscope can be selected as the discretization time interval of the filter. Considering that the measurements from the camera and accelerometer play a role in providing long-term stability, they can be updated at a low frequency. If vector or geometry measurement is not available in current sample time $t_{k}$, the corresponding correction term $\Delta \omega_{k}$ is set to zero, where *x* is *a* or *c*.

This structure of the proposed filter is the so-called “explicit version” of the nonlinear attitude observer. As we discussed in the section “Related works”, Mahony provides three versions of nonlinear attitude observer in his work: direct, passive, and explicit. The direct and passive versions depend on the algebraic reconstruction of the attitude. For MARG sensors, the sampling frequencies of accelerometer and magnetometer are the same. This will not cause any problems. However, for inertial sensors/camera combination, the difference in sampling frequency makes the algebraic reconstruction have to align with the sensor sampled in low-frequency. The consequence is that a lot of information in the high-frequency sensor is lost. On the contrary, the angle rate correction term of the explicit complementary filter in (32) is aligned with the sensor sampled in high-frequency. This is the advantage of the explicit version of the nonlinear attitude observer in handling sensors with different sample frequencies.

## 5. Evaluation

### 5.1. Experiment Setup

**Figure 2:**
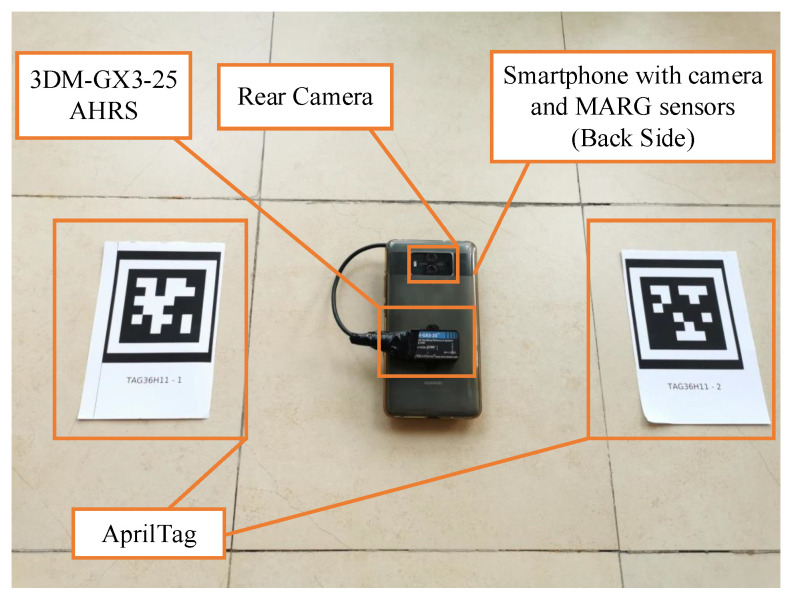
Experiment system for algorithm evaluation.

To evaluate the performance of the proposed attitude estimation algorithm, an experiment system is constructed as shown in Figure 2. A smartphone with built-in inertial sensors and a rear camera is used as the measurement equipment. Two artificial fiducials from AprilTag family Tag36H11 are placed on the ground. The 3DM-GX3-25 Attitude and Heading Reference System (AHRS) attached to the smartphone is used as the ground truth provider.

An Android application is developed to capture the data from inertial sensors and images from the camera. The sampling frequency of inertial sensors and camera are 100 Hz and 5 Hz respectively. All the data are time-stamped and stored in the smartphone’s SD card. The recorded data are processed offline on the laptop so that the different algorithms and control variables can be evaluated on the same recorded data.

To keep the AHRS from magnetic disturbance, the experiment is implemented in an outdoor environment and the magnetometer of the 3DM AHRS is re-calibrated after installation. The y-axis of the navigation frame is chosen as the magnetic north.

The intrinsic parameters and distortion parameters of the camera are pre-calibrated using the geometric method proposed in [34]. The process of extracting the normalized image coordinates of the feature points from the image is as follows. Undistort the image according to the distortion parameters. Detect the AprilTag features using the Python package pupil_apriltags. Recover the normalized image coordinates of features from the corresponding index coordinates using camera intrinsic parameters.

**Figure 3:**
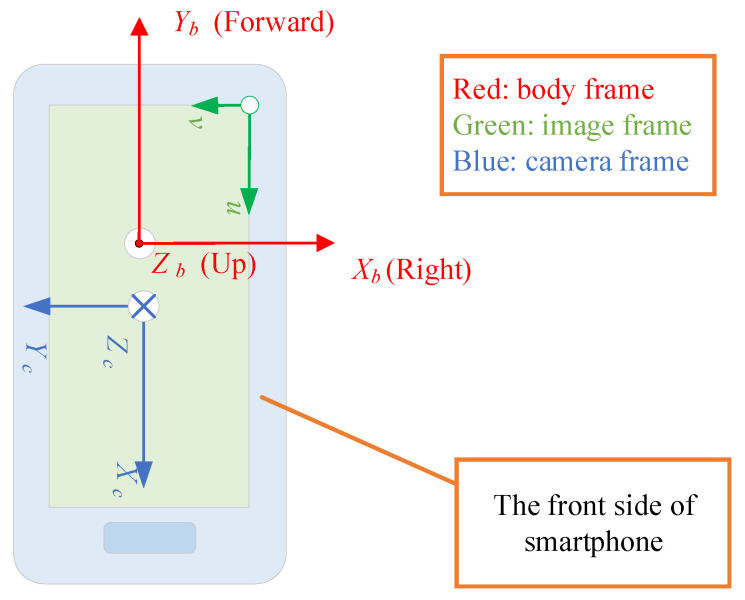
Direction relationship between the body frame image pixel frame and camera frame in experiment system.

In Section 2 and Section 3, we assume that the axis direction of the camera frame {c} is the same as that of the body frame {b}. However, in our smartphone experiment platform, the optical axis of the camera is opposite to the z-axis of the body frame. Moreover, in data collection software, the image orientation is “Landscape” relative to the smartphone. The actual direction relationship between the body frame $X_{b}$-$Y_{b}$-$Z_{b}$, image pixel frame *u*-*v*, and camera frame $X_{c}$-$Y_{c}$-$Z_{c}$ is as shown in Figure 3. Therefore, the normalized image coordinate values should be transformed to adapt to the complementary filter algorithm. The coordinate transformation matrix M is

$$M=\left[\begin{array}{ccc} 0 & -1 & 0\\ -1 & 0 & 0\\ 0 & 0 & -1 \end{array}\right].$$

Under the definition of the reference frames in Figure 3, the relationship between the normalized image coordinates of the feature points and their camera coordinates is as follows.

(33) $$p_{i}={\left[\begin{array}{ccc} \frac{P_{i,x}^{\left\{c\right\}}}{-P_{i,z}^{\left\{c\right\}}} & \frac{P_{i,y}^{\left\{c\right\}}}{-P_{i,z}^{\left\{c\right\}}} & \frac{P_{i,z}^{\left\{c\right\}}}{-P_{i,z}^{\left\{c\right\}}} \end{array}\right]}^{T}+\mu_{pi},\;i=1,2$$

This makes the third step of the initial alignment a little bit different from Section 4.3. Specifically, the “depth equations” in (25) should be replaced by (34). The attitude solutions that lead to negative $z_{1}$ and $z_{2}$ will be accepted as the initial attitude.

(34) $$C_{n}^{b}\left(P_{1}^{\left\{n\right\}}-P_{2}^{\left\{n\right\}}\right)=-z_{1}p_{1}+z_{2}p_{2}$$

Two sets of measurement data are collected to evaluate the static and dynamic performance of the proposed attitude estimation algorithm.

(1) In the static case, the smartphone keeps static in each fixed attitude about 15 s. Moreover, the smartphone in this case only makes the rotational motion between each fixed attitude without displacement relative to the navigation frame.
(2) In the dynamic case, the smartphone makes arbitrary rotation and translation movements while ensuring that the two visual labels are always within the camera’s field of view.

Representative algorithms are implemented to process the collected data for performance comparison.

(1) SINS: Attitude update algorithm of strap-down inertial navigation in [12].
(2) VIN-EKF: This is a vision-aided inertial navigation algotithm based on extended Kalman filter (EKF). The system state vector of the filter includes unit quaternion, velocity, position, gyroscope and accelerometer measurement bias. The measurement residual is computed by the measured normalized image coordinates of the feature points with the predicted normalized image coordinates in the filter. The linearized system model for the IMU error-state and the linearized measurement model about the estimates for the attitude and position of the camera are as described in [27]. The standard deviations of sensor noise are $\sigma_{gyro}=0.04{}^{\circ}/s$, $\sigma_{acc}=1\;mg$, and $\sigma_{camera}=7/f_{c}$, where $f_{c}$ is the camera focal length comes from the calibrated intrinsic parameters. Fusing camera and inertial sensors in a tightly coupled scheme is the core idea of [10] so that the vision-based visible light communication positioning system is able to continually provide location service when the number of the feature points is less than four.
(3) Proposed CF: This algorithm fuses inertial sensors and a camera using the quaternion-based discrete implementation in (28) and (32). Two gain parameters of the proposed nonlinear complementary filter are $k_{a}=0.6$ and $k_{c}=0.8$.
(4) CF-1: This algorithm also fuses inertial sensors and a camera using nonlinear complementary filter. The main difference between CF-1 and the proposed CF is that in CF-1, the measurement models of accelerometer and camera are all the vector measurements. The reference vectors are the normalized gravity vector and the normalized image coordinates of feature points in the initial frame [29]. Therefore, this is a relative attitude estimation method. To get the full attitude, the initial attitude should be known. This filter is implemented as a quaternion filter in (28), but the correction terms are all constructed using cross product of the estimated vectors and the observed vectors. There are three parameters for CF-1 when two feature points are captured in one image, that is, $k_{a}=0.6$, $k_{c1}=0.8$ and $k_{c2}=0.8$.
(5) CF-2: This algorithm fuses the gyroscope with a camera using nonlinear complementary filter. Same as the proposed CF, CF-2 also uses the camera measurements as the implicit geometry measurement. However, there is no gravity measurement in CF-2 [30]. This filter is implemented as a quaternion filter in (28), but the accelerometer correction term is zero. There is only one parameter for complementary filter when two feature points are captured in one image, that is, $k_{c}=0.8$.

The gains of the proposed CF are tuned to achieve relatively good attitude estimation results in our experimental conditions. To be fair, the gains of the other two complementary filter algorithms are set to the same constant as the proposed CF. The parameters of VINS-EKF are chosen according to the sensor measurement noise characteristics.

### 5.2. Result and Discussion

**Figure 4:**
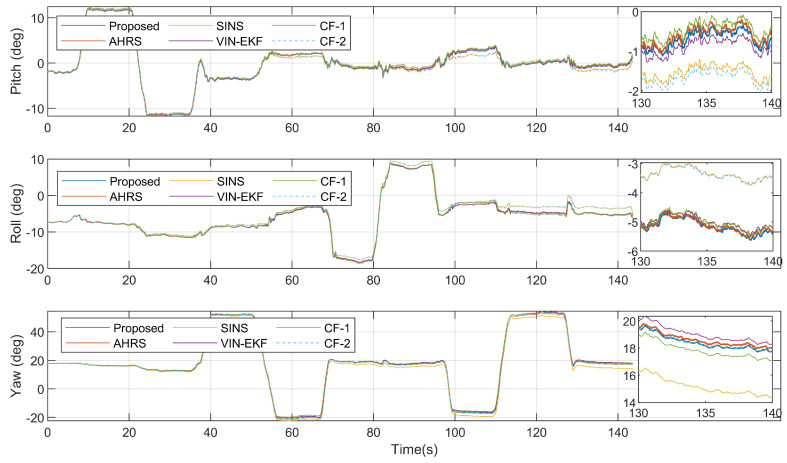
Attitude estimation results in the static case.

**Figure 5:**
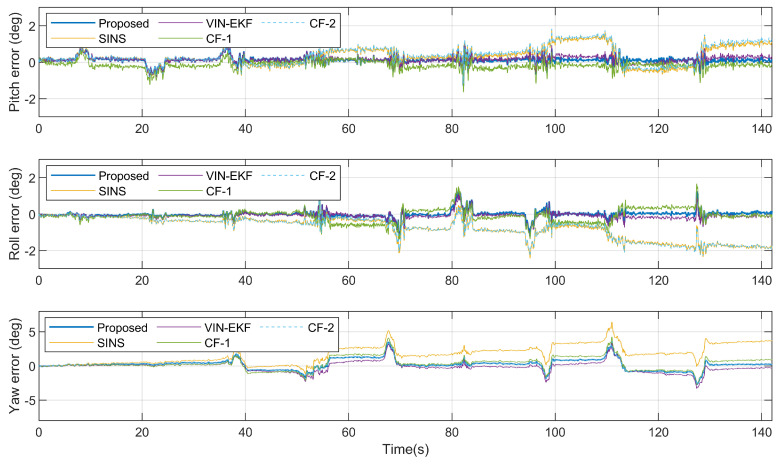
Attitude estimation error in the static case.

Figure 4 shows the attitude ground truth from 3DM AHRS, the attitude updated from the gyroscope, and the attitude estimation results from four estimation algorithms, in the static case. The attitude errors with respect to reference angles from AHRS are shown in Figure 5. To further verify the performances, Table 1 gives the root-mean-squared errors (RMSEs) of various estimation algorithms.

As can be seen from Figure 5 and Table 1, in the static case, the accuracy of the proposed algorithm is as good as that of VIN-EKF. For the CF-1, the estimation error of pitch and roll is close to that of the proposed filter, but the yaw angle error is the maximum among all the algorithms. This is due to the fact that the CF-1 algorithm corrects the gyroscope prediction error resort to vector measurement and the reference vectors of two feature points are near the gravity vector during the experiment. The result of CF-2 is just the opposite. The yaw angle error is close to the proposed algorithm, but the pitch and roll errors are almost equal to the errors of the SINS algorithm. The reason is that artificial vision fiducials are arranged on the ground, which leads to a horizontal reference vector of r in the implicit geometry measurement. Since no gravity vector or other reference vectors with vertical components are fused in the filter, the pitch and roll errors cannot converge.

**Figure 6:**
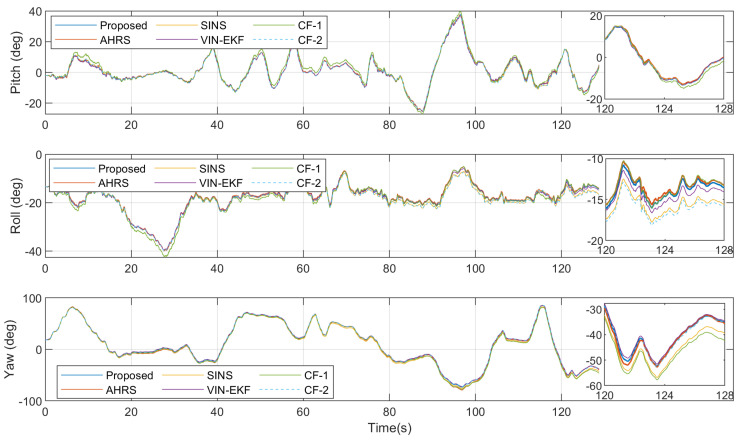
Attitude estimation results in the dynamic case.

**Figure 7:**
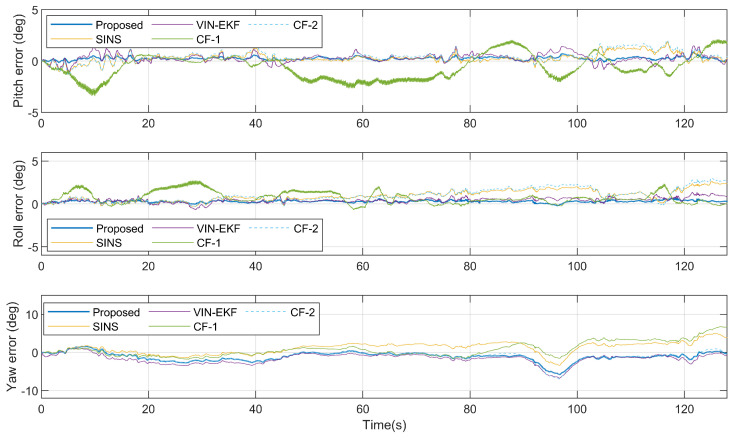
Attitude estimation error in the dynamic case.

Figure 6 shows the attitude ground truth from 3DM AHRS, the attitude updated from the gyroscope, and the attitude estimation results from four estimation algorithms, in the dynamic case. The attitude errors with respect to reference angles from AHRS are shown in Figure 7. Table 2 gives the root-mean-squared errors (RMSEs) of various estimation algorithms.

As can be seen from Figure 7 and Table 2, in the dynamic case, due to the translation motion of the smartphone, the accuracy of the VIN-EKF and CF-1 is significantly reduced. The translation motion even increases the attitude error of SINS in the early stage. In the dynamic case, the proposed algorithm offers the best performance in estimation accuracy.

For the CF-1 algorithm, the translation motion increases the error of the camera vector measurement model. The estimation accuracy of pitch and roll angle is obviously affected since the reference vector is near the vertical line. Different from the CF-1, the proposed nonlinear complementary filter fuses the implicit geometry measurement so that the position of the camera is decoupled with the attitude estimation.

When it comes to the VIN-EKF, another reason for the performance degradation that must be mentioned is the update rate of the filter. The filter propagates the state in 100 Hz and performs update in 5 Hz. Although the gravity vector constraint is implicit in the velocity equation of the system model, the innovation of this constraint becomes available only when a new image comes and the filter is updated. The estimation error of pitch and roll angle using our method is less than VIN-EKF since the gravity vector constraint is an explicit measurement and the corresponded error is corrected in 100 Hz.

**Figure 8:**
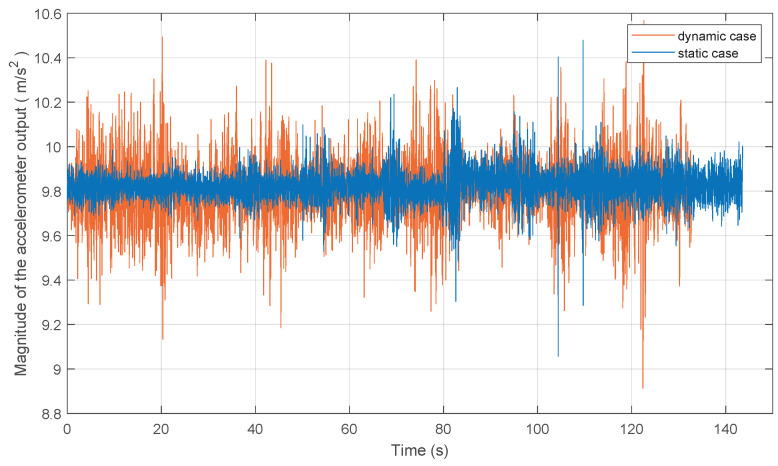
Magnitude of the accelerometer output.

Figure 8 shows the instantaneous magnitude of the accelerometer output during the static case and dynamic case.

The mean time consumption and related standard deviation of different algorithms are presented in Table 3. Here, time consumption refers to the execution time between two consecutive camera sample updates. The mean values and the standard deviation are calculated after 700 image sample updates. It can be seen that three nonlinear complementary filters show comparable time consumption. The execution time of CF-2 is the smallest since there is no accelerometer correction. The execution time of CF-2 is less than the proposed CF which means the update of vector measurement is simpler than the update of implicit geometry measurement. The execution time of VIN-EKF is the largest due to the calculation of the gain matrix and covariance matrix.

## 6. Conclusions

In this paper, we consider the attitude estimation problem of fusing camera and inertial sensors in an environment with only two pre-calibrated artificial vision fiducials. The main contributions of this paper are twofold. First, we derive an implicit geometry measurement for camera-based attitude estimation. Second, a nonlinear complementary filter with only two parameters to be adjusted is proposed to fuse angle rate measurement, vector measurement, and geometry measurement.

Compared to MARG sensor-based attitude estimation, the method in this paper doesn’t rely on a magnetometer. It provides an attractive solution for the environment with complex magnetic field distribution. Compared to camera- and gyroscope-based attitude estimation, our method still converges when there are only two feature points in the field of view. The experimental results show that our algorithm outperforms all the compared counterparts in estimated accuracy and provides competitive computational complexity.

We believe that the proposed method can potentially benefit related navigation applications. The main drawback of our method is that the designed nonlinear complementary filter only ensures locally asymptotic stability. Future work will focus on system improvements to achieve unrestricted local stability and even global stability.

## Figures and Tables

**Table 1:** RMSE of attitude angles in the static case.

Algorithm	Pitch	Roll	Yaw
CF-1	0.2701°	0.3575°	0.9912°
CF-2	0.6538°	0.9769°	0.7974°
Proposed CF	0.2195°	0.2008°	0.7977°
VIN-EKF	0.2556°	0.2160°	0.7805°

**Table 2:** RMSE of attitude angles in the dynamic case.

Algorithm	Pitch	Roll	Yaw
CF-1	1.3112°	1.0108°	2.1121°
CF-2	0.6727°	1.2696°	1.7517°
Proposed CF	0.2906°	0.3071°	1.6495°
VIN-EKF	0.5077°	0.5211°	2.1093°

**Table 3:** Mean and standard deviation of time consumption of various algorithms.

Algorithm	Mean Time	STD
CF-2	1.1162$\times 10^{-4}$ s	3.7175$\times 10^{-5}$ s
CF-1	1.4818$\times 10^{-4}$ s	2.8018$\times 10^{-5}$ s
Proposed CF	1.7010$\times 10^{-4}$ s	4.3637$\times 10^{-5}$ s
VIN-EKF	5.4172$\times 10^{-4}$ s	8.3306$\times 10^{-5}$ s
